# Identification of a Conserved Anti-Apoptotic Protein That Modulates the Mitochondrial Apoptosis Pathway

**DOI:** 10.1371/journal.pone.0025284

**Published:** 2011-09-30

**Authors:** Yu Zhang, Elisabet Johansson, Marian L. Miller, Reiner U. Jänicke, Donald J. Ferguson, David Plas, Jarek Meller, Marshall W. Anderson

**Affiliations:** 1 School of Pharmacy, University of Cincinnati, Cincinnati, Ohio, United States of America; 2 Department of Environmental Health, College of Medicine, University of Cincinnati, Cincinnati, Ohio, United States of America; 3 Laboratory of Molecular Radiooncology, Clinic and Policlinic for Radiation Therapy and Radiooncology, Clinical Center of the University of Düsseldorf, Düsseldorf, Germany; 4 Department of Microbiology, Miami University, Oxford, Ohio, United States of America; 5 Department of Cancer and Cell Biology, College of Medicine, University of Cincinnati, Cincinnati, Ohio, United States of America; 6 Division of Biomedical Informatics, Departments of Environmental Health and Biomedical Engineering, University of Cincinnati, Children's Hospital Medical Center, Cincinnati, Ohio, United States of America; 7 Department of Medicine, Cancer Center, Medical College of Wisconsin, Milwaukee, Wisconsin, United States of America; McGill University, Canada

## Abstract

Here we identified an evolutionarily highly conserved and ubiquitously expressed protein (C9orf82) that shows structural similarities to the death effector domain of apoptosis-related proteins. RNAi knockdown of C9orf82 induced apoptosis in A-549 and MCF7/casp3-10b lung and breast carcinoma cells, respectively, but not in cells lacking caspase-3, caspase-10 or both. Apoptosis was associated with activated caspases-3, -8, -9 and -10, and inactivation of caspases 10 or 3 was sufficient to block apoptosis in this pathway. Apoptosis upon knockdown of C9orf82 was associated with increased caspase-10 expression and activation, which was required for the generation of an 11 kDa tBid fragment and activation of Caspase-9. These data suggest that C9orf82 functions as an anti-apoptotic protein that modulates a caspase-10 dependent mitochondrial caspase-3/9 feedback amplification loop. We designate this ubiquitously expressed and evolutionarily conserved anti-apoptotic protein Conserved Anti-Apoptotic Protein (CAAP). We also demonstrated that treatment of MCF7/casp3-10b cells with staurosporine and etoposides induced apoptosis and knockdown of CAAP expression. This implies that the CAAP protein could be a target for chemotherapeutic agents.

## Introduction

CAAP (C9orf82) is an unannotated gene residing between 26,830,685 and 26,882,725 bp on Chr 9p harboring two alternative transcriptional start sites and six exons with a total length of 2143 bp, as well as a 3′ UTR of 1047 bp (Ensembl). We originally detected this gene by a 5′ RACE analysis when searching this region for genes related to tumorigenesis [1]. Our preliminary studies indicated several characteristics of CAAP that merited further investigation. First of all, it exhibited a high degree of evolutionary conservation, and was expressed at some level in every human tissue examined from panels of both normal (adult and fetal) and tumor tissues. Moreover, using bioinformatics approaches, one of its putative domains was predicted to share structural similarity with the death effector domain (DED). DED and the related death domain (DD) are found in a large superfamily of proteins that regulate apoptosis [2]. Therefore, we further characterized CAAP as a potential agent in apoptosis-related signaling.

Apoptotic signaling pathways are induced by activation of caspases which then cleave key protein substrates resulting in cell death [3]. Based on their structure, caspases can be divided into two classes. Caspases-2, -8, -9, and -10 contain long amino-terminal prodomains and normally function as initiators of apoptotic pathways, whereas caspases-3, -6, and -7 have only short prodomains and function as effectors of cell death [4]–[8]. The activation of the initiator caspase-9 in the intrinsic mitochondrial apoptosis pathway involves BH3 proteins of the Bcl-2 family that function as monitors of cellular damage. In response to cellular damage, these proteins promote activation of the pro-apoptotic activities of Bax and Bak, inducing the release of cytochrome c, and subsequent formation of the apoptosome, which is a multi-subunit caspase scaffold that activates the caspase-9-dependent apoptotic pathway [9]–[11]. In the death receptor-mediated apoptosis pathway, a protein complex recruiting the Fas-associated protein with a death domain (FADD), and procaspase-8 and/or -10 is called the death-inducing signaling complex (DISC) [12]. The procaspases-8 and -10 in the DISC are activated by oligomerization followed by proteolytic self-processing enabling them to activate downstream effector caspases including caspase-3 [4]. Recent studies of the mitochondrial apoptosis pathway demonstrate that caspase-8 and -10 can also be activated downstream of the mitochondria by caspase-3, indicating the existence of so-called amplification loops where caspase-8 or -10 activate caspase-9 and -3 [13]–[18].

In this context, it should be noted that activated caspase-8 and -10 can also proteolytically activate pro-apoptotic Bcl-2 family member Bid generating tBid [17], [18]. tBid causes the release of mitochondrial cytochrome c resulting in the activation of caspase-9 which can further enhance caspase-3 activity to complete the apoptotic process [19]–[21].

To determine whether and to what extent CAAP is involved in the regulation of apoptosis, we examined caspase activation and apoptosis signaling in the presence and absence of CAAP in several tumor models. Our study revealed that CAAP exerts a prominent anti-apoptotic function that critically depends on the presence of caspases-3 and -10. In addition, we demonstrated that treatment of MCF-7/casp3-10b cells with staurosporine and etoposide triggered knockdown of the CAAP expression concurrent with the induction of apoptosis. These data suggest that CAAP may be a target site for chemotherapy since it does not require siRNA to knockdown the expression of this anti-apoptotic protein.

## Materials and Methods

### Cell line and cell culture

The human lung carcinoma cell line A-549 was obtained from ATCC (Manassas, Virginia) grown at 37°C under 5% CO_2_ in RPMI-1640 medium supplemented with 10% FBS and antibiotics. MCF-7 breast carcinoma cells were maintained in RPMI-1640 supplemented with 10% fetal bovine serum and antibiotics. MCF-7/casp3 cells stably expressing caspase-3, MCF-7/casp-10b stably expressing caspase-10b and MCF-7/casp3-10b stably expressing both caspase-3 and caspase-10b were maintained in the same medium supplemented with either 400 µg of neomycin/ml (Fisher Scientific) [MCF-7/casp3], 200 µg of hygromycin/ml (Santa Cruz Biotechnology) [MCF-7/casp-10b] or a combination of both antibiotics [MCF-7/casp3-10b].

### Plasmid DNA

Myc-FADD-DN (dominant-negative form of FADD) was from Keyclone Technologies. Cincinnati. OH.

### siRNA Preparation and Transfection

siRNA probes were synthesized using Silencer® siRNA Construction Kit from Ambion (Austin, Texas). The siRNA Target Finder (http://www.ambion.com/techlib/misc/siRNA_finder.html) was used to select target sequences, and sequences selected were analyzed using BLAST (http://www.ncbi.nlm.nih.gov/BLAST/) to minimize off-target knockdown effects. Two target sequences denoted as si48 and si67 were selected in CAAP:

si48: 5′-AACCTGAAGGTTTGGAATTAA-3′

si67: 5′-AAGTGTGAATGAGATTCTAGG-3′

The non-targeting siRNA negative control (Ncontrol) (D-007206-13-20) and the sicasp-8 were purchased from Dharmacon Research Inc:

sicasp-8: 5′AA-GGGUCAUGCUCUAUCAGAU-dTdT-3′

Transfection of A549 cells or MCF-7, MCF-7/casp3, MCF-7/casp10, and MCF-7/casp3-10b cell lines was performed according to the procedure used by Sharp et al (2005) [22]. Cells were transfected at a confluency of 85–90%. Prior to transfection, regular medium was replaced with low serum (4% FBS) medium. The cells were transfected with siRNA using Lipofectamine (LF 2000) at a ratio of 1∶3 siRNA (µg): Lipofectamine (µl). The final concentration of siRNAs was 43 or 65 nM for A-549 cells and 22 nM for MCF cells. The cells were incubated with the siRNA-Lipofectamine 2000 complex overnight. For A549 cells, 24 hrs after transfection the low serum medium was replaced with normal medium (10% FBS) without antibiotics and 48 hr after transfection floating and adherent cells were harvested and prepared for analysis of apoptosis. Floating and adherent MCF cells were harvested 24 hours after transfection, and prepared for analysis of apoptosis.

### Reverse transcription and PCR

For RT-PCR, total RNA was isolated using TRIzol® reagent from Invitrogen (Carlsbad, California) according to manufacturer's instructions. First-strand cDNA was synthesized by reverse transcription of 1 µg total RNA from cell lines for 60 minutes at 42°C using random primers and 20 units AMV reverse transcriptase in a total volume of 20 µl.

### Analysis of CAAP expression

For screening CAAP mRNA expression in normal, fetal, and tumor tissues, MTC Multiple Tissue cDNA panels were obtained from Clontech (Mountain View, California). The primers used for amplification of the main splice variant (CAAP) were DS93SPLJ1/2F (5′-CTCCTTGCAGCAGGAAACTA-3′) which spans the boundary between exon 1 and exon 2, and DS93E2R2 (5′-ATGACACAGTCAGGTCCAGT-3′) which is located in exon 2. For amplification of the alternative splice variant (CAAP-ASV1) the primers used were DS93ASVF2 (5′-GTCCGTCCTTGAGGTTCATGT-3′) and DS93E2R2. PCR was performed in 10 µl volumes containing 0.5 µM of each primer, 1.5 mM magnesium chloride, 200 µM of each dNTP, and 0.25 units Platinum Taq DNA polymerase (Invitrogen). Standard PCR analysis was performed. Twenty PCR cycles were used for each tissue. A human control tissue (Clontech) was analyzed in each of the panels and results shown in the last column of each panel.

### Quantitative PCR to assay suppressed CAAP expression

For the determination of a successful knockdown of CAAP mRNA expression in A549 cells after transfection with si48, si67, and Ncontrol siRNAs, total RNA was isolated and first-strand cDNA was synthesized by reverse transcription of 1 µg total RNA from cell lines for 60 minutes at 42°C using random primers and 20 units AMV reverse transcriptase in a total volume of 20 µl. For amplification of CAAP the primers used were DS93SPLJ1/2F and DS93E2R2 as defined above. As a control, GAPDH was amplified using the primers 5′-CAGGAGGCATTGCTGATGAT-3′ and 5′-AGCGAGATCCCTCCAAAATC-3′. Quantitative PCR was carried out in 25 µl reactions containing 12.5 µl iQ SYBR GREEN supermix (BioRad, Hercules, CA), 0.2 µM of each primer and 10 ng of cDNA. The reactions were run using the Realplex Mastercycler (Eppendorf) and the PCR program included initial denaturation at 94°C for 10 minutes and then 50 cycles of 30 s at 94°C, 30 s at 56°C, and 45 s at 71°C. Cycle number and percent knockdown of CAAP mRNA expression by siRNAs were analyzed by using Eppendorf Mastercycler ep Realplex software.

### Apoptosis Assays

To quantify apoptosis for both the adherent and floating cells, we used the M30 FACS assay and a morphometric assay, respectively. Media from plates were collected 24 hrs after transfection of MCF cells and 48 hrs after transfection of A549 cells. After cell clumping was dispersed by pipeting, floating cells were counted in aliquots of the media to determine the total number of floating cells. The media was then centrifuged at 1200 rpm for 5 minutes to obtain a pellet of floating cells. The cells were resuspended in a small quantity of phosphate buffered, isoosmolar, 2.5% gluteraldehyde/2% paraformaldehyde fluid and placed in a conical Beem capsule and centrifuged at 1000 g for 5 minutes. Cells were fixed overnight, post fixed in 1% osmium tetroxide for 2 hr, dehydrated through graded ethanols, propylene oxide and embedded in Spurr's resin. One micron thick sections were made in at least two levels within the cell pellet, and were stained with toluidine blue for light microscopy. Cells were counted at 1250×. The mean number of cells counted per group was approximately one thousand, except for Ncontrol treatment where all cells were counted. The fraction of floating cells which were apoptotic was determined from these data.

Immediately after media was removed as described above, adherent cells were washed with cold PBS and trypsinized. After cell clumps were dispersed by pipetting, trypsinized cells were counted in the PBS solution to determine the total number of adherent cells. Cells were washed in PBS and fixed in ice-cold methanol for 30 min at −20°C. Fixed cells were stained with M30 CytoDEATH antibody according to manufacturer's instructions. The antibody M30 CytoDEATH (Roche Applied Sciences) recognizes a specific caspase cleavage site within cytokeratin 18 that is not detectable in the native keratin. Stained cells were resuspended in PBS to 2×10^5^ cells/ml. After removal of clumping by pipetting, FACS analysis was run on a BD FACS ARIA instrument. At least 10^4^ cells were analyzed for each sample. The fraction of adherent cells which were apoptotic was determined from the M30 FACS assay data.

Since we counted the total # of floating cells and adherent cells, the % of floating and adherent cells was determined, and thus,

Percent of apoptosis was calculated as following:

Percent of apoptosis = % of floating cells×fraction of floating cells that are apoptotic+% of adherent cells×fraction of adherent cells that are apoptotic. The percentage of floating cells that were apoptotic was always greater than 95% and thus we used this number to calculate apoptosis.

### Caspase activity measurement

The day before transfection 3.0×10^5^ A-549 cells or 4.0×10^5^ MCF cells were plated in 60 mm dishes. After 24 hrs A549 cells were transfected with either Ncontrol 65 nM or si67-65 nM siRNA, MCF cells were transfected with either Ncontrol 22 nM or si67-22 nM siRNA and 16 or 24 hrs after transfection floating and adherent cells were combined and prepared for caspase activity measurements. Caspase activities were measured by using the caspase colorimetric assay kit from BioVision by following manufacturer's instructions. Briefly, cell lysates (50–100 µg protein in 50 µl lysis buffer) were incubated with 200 µM of colorimetric caspase-3 substrate DEVD-pNA, caspase-8 substrate IETD-pNA, caspase-9 substrate LEHD-pNA and caspase-10 substrate AEVD- pNA in 50 µl of 2× reaction buffer containing 10 nM DTT at 37°C for 2 hrs, and samples read at 405 mm in a microtiter plate reader.

### Effect of FADD-DN on apoptosis induced by CAAP knockdown or TRAIL

The day before transfection, 3×10^5^ A-549 cells were plated in 60 mm dishes. The next day empty vector or FADD-DN were transfected into cells using LF 2000. After 24 hrs proteins were isolated from the cells as described below.

A-549 cells (3×10^5^) were plated in 60 mm dishes and after 24 hrs cells were transfected with empty vector or FADD-DN. After 24 hrs, cells were transfected with si67 (65 nM) or treated with TRAIL (35 ng/ml). Floating and adherent cells were harvested 24 hrs after transfection, and prepared for analysis of apoptosis.

### Western Blot Analysis

Cells were washed with cold PBS and lysed with RIPA buffer in the presence of protease inhibitors, sonicated for 15 seconds, and then centrifuged at 12,000× g at 4°C for 20 min. 35 µg of cellular protein (quantitated by using the BCA protein assay from Pierce) was loaded onto 4–12% NuPAGE Bis-Tris gradient gels (invitrogen), electrophoresed and electrotransferred onto PVDF membrane according to manufacturer's instruction. Membranes were then blocked with 5% milk TBS-T for 1 hr at room temperature. Proteins were detected with the following antibodies: rabbit anti-caspase 3 pAb ( BioMol), mouse anti-caspase 8 mAb, rabbit anti-β-actin pAb and rabbit anti-caspase-9 pAb (Cell Signaling Technology), mouse anti-caspase-10 (MBL med and BioLab), mouse anti-Human FADD mAb (BD Transduction Laboratories), rabbit anti-CAAP pAb (Quality Controlled Biochemicals, Hopkinton, MA), rabbit anti-Bid pAb, and mouse anti-PARP mAb ( BD Phararmingen). Membranes were incubated with primary antibody overnight at 4°C and followed by incubation with HRP-conjugated secondary antibodies (Cell Signaling Technology). Immunoreaction bands were visualized by using enhanced chemiluminescence (Perkin Elmer).

### Treatment of MCF-7/casp3-10b cells with chemical agents

MCF-7/casp3-10b cells were treated with either staurosporine (STS) or etoposide. STS treatment: MCF-7/casp3-10b cells were treated at dose of 1.0 µM for 4, 6, and 8 hrs and then western blot analysis was performed to examine CAAP expression and cleavage of caspase-3 and -9. Etoposide treatment: MCF-7/casp3-10b cells were treated at dose of 100 µM and 500 µM for 24 hrs and then harvested for western blot analysis of CAAP expression and cleavage of PARP and procaspase-10 expression.

## Results

### Genomic organization and evolutionary profile of CAAP

**Figure 1:**
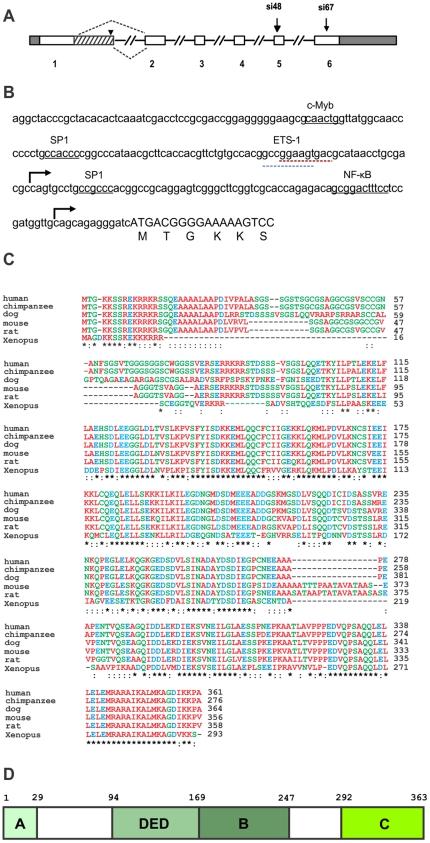
Genomic organization and evolutionary profile of CAAP. (**A**) Unfilled bars represent coding sequence, and grey bars untranslated regions. The hatched bar shows the extension of the first exon that creates an alternative transcript. The arrowhead indicates the stop codon in CAAP-ASV1. Target locations of siRNA sequences are indicated by arrows. (**B**) The nucleotide sequence of the promoter region of CAAP harbors several transcription factor binding sites (underlined). Bent arrows show transcriptional start sites according to ENSEMBLE. See the text that describes the sequence with dashed red (and blue) underline. **Evolutionary profile of CAAP**. (**C**) Multiple sequence alignments were obtained using the ClustalW server: asterisks indicate conserved (within the orthologs considered here) positions, whereas colons indicate positions conserved in all but one species included in the figure: amino acid residues are colored according to their physicochemical properties, as defined at http://www.ebi.ac.uk/Tools/clustalw/color_frame.html. (**D**) Schematic representation of the overall structure of CAAP with putative domains shown as shaded boxes: the N terminal conserved basic domain, Box A; DED domain, Box DED; central conserved domain adjacent to DED, Box B; and a conserved C terminus domain, Box C. Two variable and relatively unstructured domains are represented by white boxes.

CAAP is located on chromosome 9p, band 21.2, and consists of six exons spanning over 51.5 kilobases (Fig. 1A). 5′-RACE experiments indicated that CAAP has an alternative splice variant (CAAP-ASV1) with an extended first exon at a canonical splice donor 154 bp downstream of the splice donor in the prototypic first exon (Fig. 1A). This splicing event was documented in dbEST, and 15 of the 140 mRNA and EST sequences listed in the Unigene cluster for CAAP contained the additional part of the first exon. CAAP-ASV1 contains a stop codon at the end of the extended first exon, which results in a truncated protein of 133 amino acids in length. Target locations of siRNA sequences are indicated by arrows (Fig. 1A).

The 5′-end and promoter regions of the gene are GC rich and do not contain a TATA-box near the presumed transcription start site. The promoter region of CAAP contains several transcription factor binding sites including NF-ĸB, cMyb, SP1, and ETS-1. A search of the TRANSFAC database showed that the promoter region contains a 10-bp sequence (GGAAGTGACG: dashed red underline) (Fig. 1B) that is also present in the promoter of the retinoblastoma gene, where it constitutes a cis-acting element susceptible to negative regulation by the tumor suppressor p53 [23]. Partly overlapping with the p53 control element is a motif (GCCGGAAGT: dashed blue underline) for binding of the Ets1 oncogene (Fig. 1B).

Multiple alignments show that CAAP contains four regions that are highly conserved among mammalian species including chimpanzee, dog, mouse, and rat as well as in more distant species like *Xenopus laevis* (Fig. 1C). These four regions are: 1) a short region at the N-terminal end including amino acid (aa) residues 1 to 29 that is rich in basic residues; 2) a helical domain between residues 94 to 169 that is predicted to share structural similarity (with very low sequence homology) with the DED; 3) a region adjacent to the putative DED between residues 170 to 247 that contains two predicted helices; and 4) a C-terminal domain between residues 292 to 361 which is highly conserved in all mammalian species examined as well as in *Xenopus*, and that is predicted to also contain two well-defined helices. The four distinct conserved domains are shown as shaded boxes in Fig. 1D. On the other hand, the predicted translation product of CAAP-ASV1 only contains the orthologous region A, as the region of the protein resulting from the extension of the first exon is not conserved among mammalian species. The function of this splice variant will not be examined in this study.

### Structural analyses of CAAP

**Figure 2:**
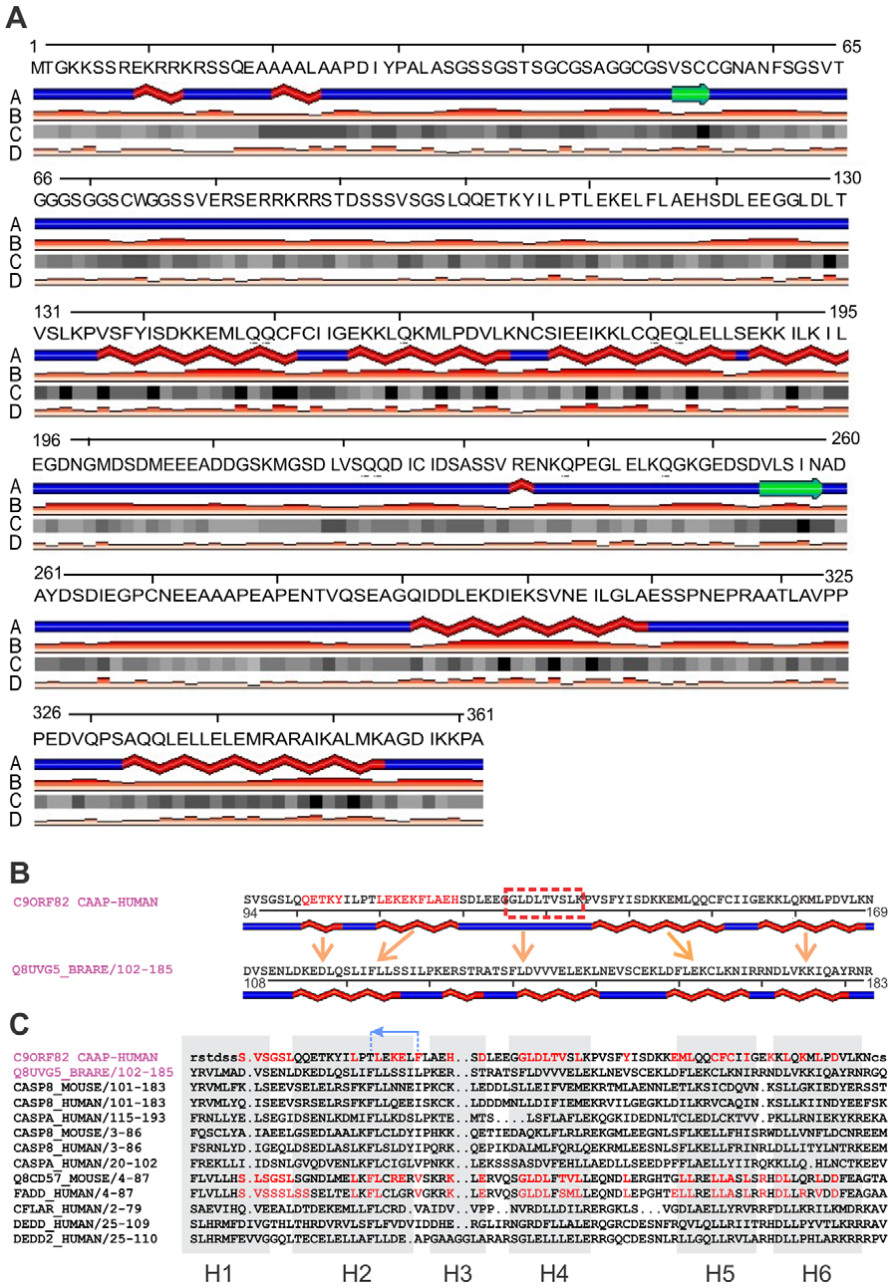
Overall structure of CAAP. (**A**) SABLE (http://sable.cchmc.org) was used to predict the structure of CAAP. The amino acid sequence, predicted secondary structures (with helices shown as red braids, beta strands as green arrows, and loops as blue lines) (row A), the confidence of secondary structure predictions at each position (row B, the higher the bar the more confident the prediction), predicted solvent accessibilities (row C, black boxes corresponding to fully buried positions, and shades of grey representing the degree of solvent exposure), and the confidence bars for the solvent accessibility prediction (row D, the higher the bar the more confident the prediction), are shown in the respective rows in the figure. **Alignment of the central CAAP domain.** (**B**) FFAS alignment of the central CAAP domain and a DED family representative Q8UVG5 (also indicated in blue in Fig. 2C). The SABLE and PsiPRED (http://bioinf.cs.ucl.ac.uk/psipred/) predicted helices (the latter highlighted using red font for amino acid residues) within the central CAAP domain are shown as red braids. As can be seen from the Figure, they are qualitatively consistent with those of the DED matching domain shown for comparison below. However, due to low homology, FFAS likely misaligns some of the helical regions, as indicated by arrows between predicted and observed helices (the latter also shown in the context of multiple alignment in Fig. 2C); **Alignment of the DED domains** (**C**) Multiple alignment of DED domains from several members of the DD superfamily, superimposed with the approximate position of canonical six helices of DD [2], which are shown as gray boxes. Note that CAAP sequence, which is shown on top (using FFAS alignment into Q8UVG5), is consistent with the range of substitutions observed within the family, and also that it appears to be most consistent with its FADD members (human and mouse), as indicated in red to highlight several conserved motifs. Note also, that several functionally relevant motifs, such as FLAEH (residues 115–119) or KxL (residues 157–159), appear to be misaligned in the automatically generated FFAS alignment.

Sequence alignment, secondary structure prediction, and fold recognition methods were used to characterize CAAP and identify its putative homologs. Simple BLAST alignments did not result in significant matches into annotated protein families. Subsequent Psi-BLAST iterations revealed only weak sequence similarity to multiple (mostly helical) protein families. To map putative domains in CAAP and to enhance subsequent fold recognition searches, we used PsiPRED and SABLE to derive consensus secondary structure, as well as solvent accessibility predictions. According to these predictions, CAAP contains two putative globular regions, one located approximately between residues 90 and 250 and another between residues 290 and 360, comprising several helices with multiple sites predicted to be fully buried in the hydrophobic core of these putative globular domains (Fig. 2A). Although PsiPRED predictions are largely consistent with those obtained by SABLE (which are shown in Fig. 2A), there are several additional helical segments predicted by PsiPRED between residues 90 and 130, which are highlighted in Fig. 2B. On the other hand, the N-terminus domain which coincides with the first exon is consistently predicted to be largely unstructured with several putative secondary structure elements. It should be noted that despite the presence of some hydrophobic fragments, CAAP is overall strongly hydrophilic and predicted to be a soluble protein.

Several fold recognition methods were used to help identify structural homologs by threading the primary sequence of CAAP through databases of known protein structures or evolutionary profiles of protein families. The well benchmarked FFAS server [24] identified a death effector domain (DED) as the best match for CAAP in the PFAM database [25]. The observed and predicted helical segments were qualitatively consistent, supporting the prediction of a 6 alpha-helical bundle between amino acids 94 and 169 (Fig. 2B). In addition, several functionally important charged as well as hydrophobic sites observed in DED of FADD (human and mouse) were conserved in CAAP, as highlighted in Fig. 2C. This indicates that not only structural, but also functional similarity could exist between the central domain of CAAP and other DED proteins.

### Ubiquitous Expression of CAAP in tissues and cell lines

Expression of CAAP was tested by PCR on commercially available primary tissue cDNA panels. It was found to be expressed in all human tissues examined (Fig. S1). In normal tissue, expression appeared to be higher in pancreas, spleen, testis, kidney, and liver, whereas brain and leukocytes exhibited only a low expression of CAAP. We also screened a variety of primary tumors and all were found to express CAAP. Moreover, CAAP was also expressed in all fetal tissues examined, with highest expression in fetal lung. An almost identical expression pattern was also found for the splice variant CAAP-ASV1 (Fig. S1). These data may reflect relative expression differences and not quantitative expression of CAAP in these tissues. In addition, as determined by RT-PCR, one immortalized normal human bronchioepthelial cell line as well as fourteen lung tumor cell lines were also found to be CAAP positive (data not shown), indicating that CAAP is a ubiquitously expressed protein.

### Knockdown of CAAP results in caspase activation and apoptosis

**Figure 3:**
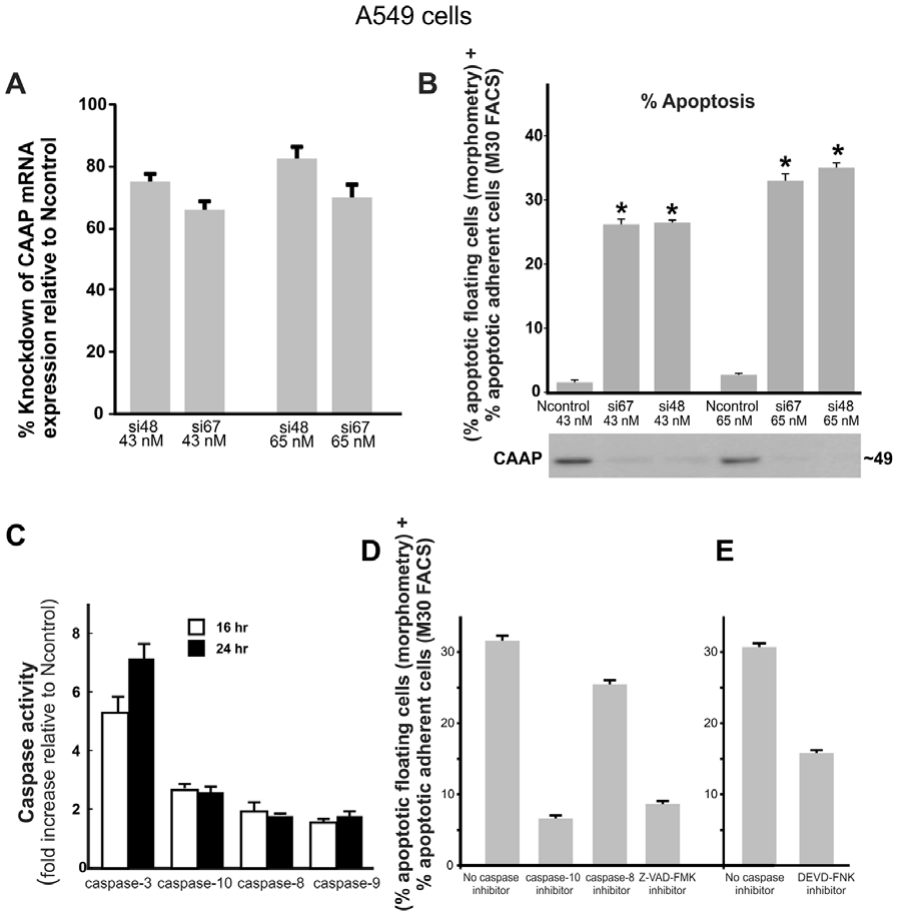
Knockdown of CAAP induces caspase-dependent apoptosis in A549 cells. (**A**) The expression levels of CAAP in lung cancer A-549 cells were knocked down by the siRNA's, si67 and si48 relative to a non-targeting control siRNA at the indicated concentrations. (the higher the bar the more confident the prediction). (**B**) Determination of apoptosis in A-549 following transfection with the two CAAP siRNAs si67 and si48 compared to the apoptosis in the non-targeting control siRNA at the indicated concentrations. (**C**) Determination of the fold increase in caspase-3, -8, -9 and -10 activities following CAAP knockdown by si67 (65 nM) compared to the Ncontrol siRNA (65 nM). (**D**) Determination of apoptosis in A549 cells 24 hours following CAAP knockdown by si67 in the absence or presence of 75 µM of IETD-FMK (caspase-8 inhibitor), AEVD-FMK (caspase-10 inhibitor), or Z-VAD-FMK, a pan-caspase inhibitor. (**E**) Determination of apoptosis in A549 cells 24 hours following CAAP knockdown by si67 in the absence or presence of DEVD-FMK (caspase-3 inhibitor).

To examine the role of CAAP in apoptosis signaling, we silenced expression of the CAAP gene in A-549 lung carcinoma cells by two independent siRNA duplexes (si67 and si48). Compared to a non-targeting siRNA pool (Ncontrol), both siRNAs produced an efficient knockdown of CAAP mRNA expression at both concentrations (43 nM and 65 nM) employed (Fig. 3A). Following transfection of A-549 cells with si48, si67 or siNcontrol, floating (Fig. S2) and adherent cells were harvested to analyze apoptosis induction as described in the Methods section. Percent of apoptosis after treatment with si48 was 27% and 35% at 43 nM and 65 nM dosages respectively, and treatment with the si67 siRNA induced apoptosis values of 28% and 33% with the two dosages employed, respectively (Fig. 3B). Thus, compared to the siNcontrol treatment, there is an obvious statistical difference in the extent of apoptosis in cells treated with si48 or si67 RNAi, p<0.001. The CAAP protein was also knocked down after siRNA treatment as shown in the panel under the apoptosis data (Fig. 3B). Based on these results, we used only the si67-65 nM probe for most of the remaining studies.

Cell lysates from A-549 cells transfected with the si67-65 nM or Ncontrol RNAi were also used to determine the activation of caspases-3, -8, -9 and -10. At 24 hrs after transfection, there was a 7.1-fold increase in caspase-3 activity for the substrate DEVD-pNA, a 2.6-fold increase in caspase-10 activity for the substrate AEVD-pNA, and a 1.8-fold increase in both caspases-8 and -9 activities for the IETD-pNA and LEHD-pNA substrate respectively (Fig. 3C). Similar results were obtained 16 hrs post transfection (Fig. 3C), suggesting that these caspases are involved in apoptosis induced by knockdown of CAAP expression.

Next, several fmk-peptide caspase inhibitors were tested to determine which caspases were required for apoptosis induced by knockdown of CAAP expression (Fig. 3D). Both, the pan-caspase inhibitor Z-VAD-FMK as well as the caspase-10 inhibitory peptide AEVD-FMK inhibited apoptosis by approximately 80%, whereas IETD-FMK that preferentially targets caspase-8, blocked cell death by only 19%. Fig. 3E shows that the DEVD-FMK caspase-3 inhibitor blocked cell death by 50%. These data suggest that apoptosis induced by the knockdown of CAAP depends mainly on caspase-10 and caspase-3.

**Figure 4:**
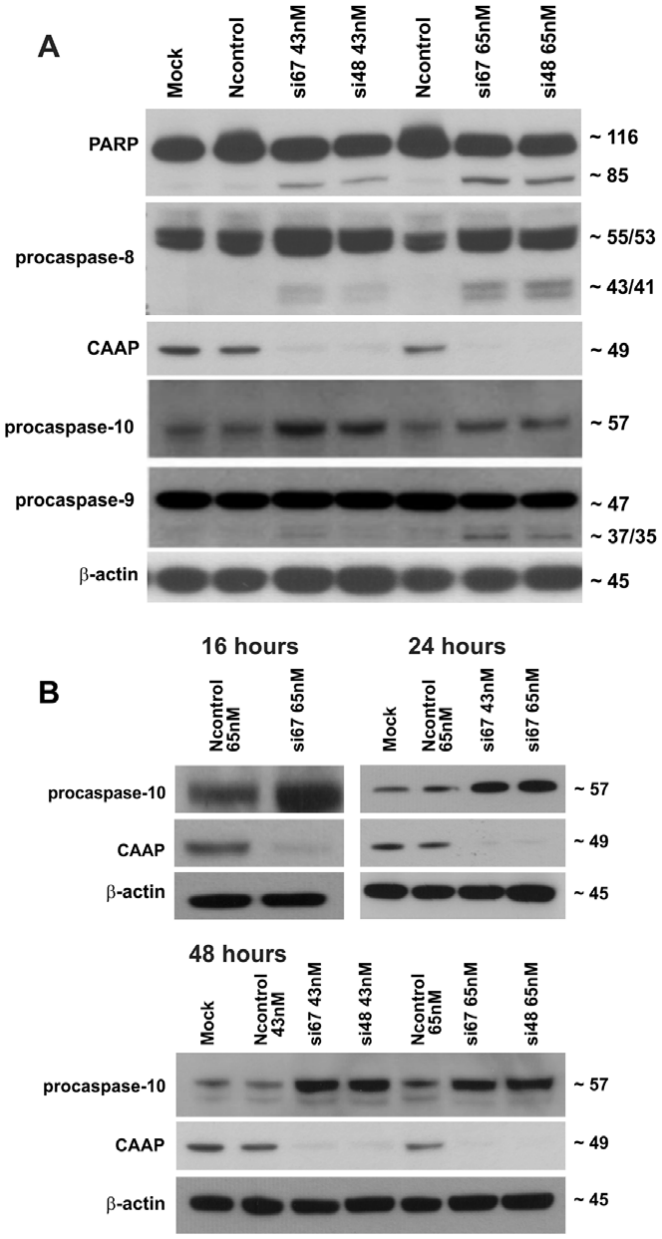
Knockdown of CAAP induces caspase processing, cleavage of PARP and results in increased expression of caspase-10. (**A**) Determination of caspase processing and PARP cleavage by Western blot analyses of floating and adherent A-549 cells 48 hours post transfection with si67 and si48 at dosages of 65 nM and 43 nM. The p85 cleavage fragment of PARP after treatment with the siRNAs and the activation of procaspase 9, as shown by the 37/35 fragments, and caspase-8 as shown by the 43/41 fragments, are consistent with the fold increase of Caspase-3, -8, and -9 activities shown in Fig. 3C and discussed in the text. Comparing the expression levels of procaspase 10 in the mock and Ncontrols to the levels in the si67 and si48 lanes implies that the expression of procaspase 10 is increased after RNAi knockdown of CAAP. A representative experiment out of three is shown. (**B**) Western blot analyses for the status of CAAP and procaspase-10 in floating and adherent A-549 cells 16 h, 24 h or 48 h post transfection with either Ncontrol or si48 or si67 siRNA. Probing the membrane with a ß-actin antibody served as a loading control. Procaspase-10 protein levels were reproducibly higher following knockdown of CAAP.

Since proteolytic activation of caspases is an important step in caspase-dependent apoptotic cell death, we examined processing of caspases-8, -9, and -10 and PARP, and a substrate of caspase-3, by Western blot analyses. Cleavage of the 116 kDa PARP proteins to the 85 kDa fragment was detected in A549 cells after knockdown of the CAAP protein with si67 and si48 RNAi at both dosages (Fig. 4A), supporting our hypothesis that knockdown of CAAP induces a caspase-dependent apoptosis program. This result is also consistent with the observed seven fold increase of caspase-3 activity as demonstrated in Fig. 3C.

We also observed processing of the 55 kDa procaspase-8 into the intermediate 43/41 kDa fragments, and the 47 kDa procaspase-9 was cleaved into its intermediate 37/35 kDa fragments (Fig. 4A). Although processing of these initiator caspases was relatively weak, these events were reproducibly observed with both doses of the CAAP siRNAs si67 and si48 and are also consistent with the fold increases of their activities (Fig. 3C). Interestingly, the expression level of procaspase-10 was significantly increased after CAAP knockdown (Fig. 4A). To verify this surprising observation, we compared the expression levels of procaspase-10 after silencing of CAAP expression at different time points. The results presented in Fig. 4B clearly demonstrate that at all time points examined the levels of procaspase-10 significantly increase following CAAP knockdown, but remain unchanged in the presence of the Ncontrol siRNA. These data suggest that CAAP might be involved in the regulation of procaspase-10 expression.

### Apoptosis induced by CAAP does not depend upon death receptor pathway

**Figure 5:**
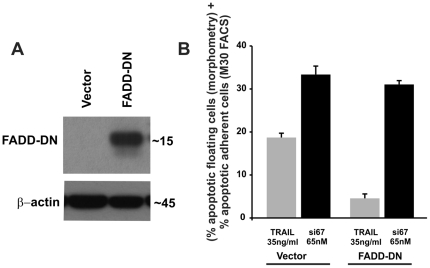
Apoptosis induced by CAAP knockdown proceeds independently of death receptor pathway. (**A**) Western blot analysis for expression of dominant-negative FADD (FADD-DN) in A-549 cells that were transfected with an empty vector or the FADD-DN construct. β-actin expression was determined as a control for loading. (**B**) Determination of apoptosis of A-549 cells that were transfected with empty vector or FADD-DN. After 24 hrs cells were transfected with si67 or treated with TRAIL (35 ng/ml). The percentage of apoptotic cells was determined after an additional 24 hrs. Apoptosis values for the si67-65 nM transfected cells or the TRAIL treated cells are expressed relative to the amount of apoptosis induced in the vector- or FADD-DN transfected cells. These data demonstrate that FADD-DN overexpression blocks TRAIL induced apoptosis but not apoptosis induced by CAAP knockdown.

To determine whether apoptosis induced by RNAi knockdown of CAAP requires the death receptor pathway, we transiently expressed a C-terminal deleted dominant-negative mutant of FADD (Fig. 5A). As expected, expression of FADD-DN protected A-549 cells from apoptosis induced by the death receptor ligand TRAIL, but not from apoptosis induced by RNAi knockdown of the CAAP gene (Fig. 5B). LF 2000 was used for transfection. The efficiency was not known since the vector does not contain a fluorescence tag. However, the data in Fig. 5B suggest an efficient transfection, as the response to TRAIL in all cells resembled vector control upon expression of FADD-DN.

### Apoptosis induced by the knockdown of CAAP critically depends upon the simultaneous presence of caspase-3 and -10

To investigate the role of caspase-10 in the apoptosis pathway induced by knockdown of CAAP, we utilized MCF-7 breast cancer cells that express neither caspase-3 nor caspase-10 [26], [27]. In addition, three derivative cell lines that stably express either caspase-3 (MCF-7/casp3), caspase-10b (MCF-7/casp-10b), or both caspases (MCF-7/casp- 3-10b) were used.

**Figure 6:**
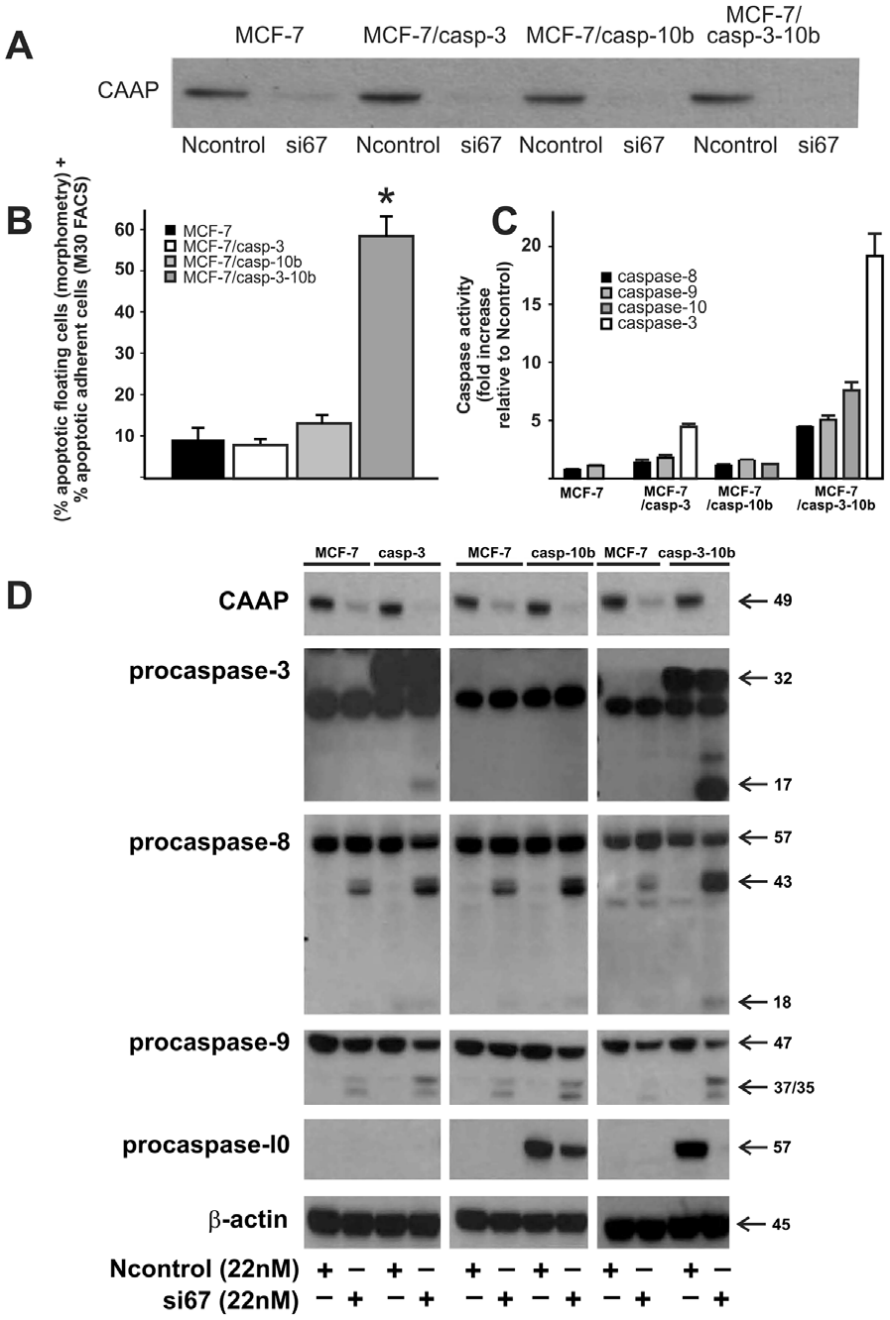
Caspase-3 and -10 are required for apoptosis induction by the knockdown of CAAP. (**A**) A western blot demonstrates that CAAP protein expression is reduced in all four of the MCF-7 cell lines after transfection of si67. (**B**) Bar graph shows the total percent of apoptosis induced by knockdown of CAAP as calculated according to the description in the Methods section. Each bar equals mean apoptosis ± SEM, from four experiments. Asterisk denotes a statistically significant difference between MCF-7/casp3-10b cells and the other MCF-7 cell lines which implies that caspase-3 and -10 are required for apoptosis induction by knockdown of CAAP (P<0.0001 according to Student's t-test). (**C**) Determination of caspase-3, -8, -9 and-10 activities in the four MCF-7 cell lines 24 hours post transfection with si67 at 22 nM compared to Ncontrol at 22 nM. Each bar equals mean fold increase ± SEM from three experiments. (**D**) Western blot analyses for the determination of caspase processing in the four MCF-7 lines. Twenty-four hrs after transfection, both floating and adherent cells were harvested for Western blot analysis. We compared each of the three derivative cells with the parent MCF-7 cells. Note that procaspase-3 is at 32 kDa and the band below is unknown. Cleavage fragments were only detected in lanes in which CAAP expression was knocked down by the si67 probe. One representative experiment out of three is shown.

Interestingly, although expression of CAAP was efficiently knocked down in all MCF-7 clones (Fig. 6A), significant apoptosis was only observed in MCF-7/casp3-10b (Fig. 6B) cells. Here, 60% of the cells were found to be dead, whereas in the other three cell lines only 8 to 13% of the cells appeared apoptotic, indicating that both caspase-3 and –10 are required for apoptosis induced by knockdown of CAAP. Consistently, a significant increase in caspase activities could only be detected in MCF-7/casp3-10b cells, but not in the parental MCF-7 cells or in the derivative line expressing caspase-10b (Fig. 6C). On the other hand, MCF-7/casp3 cells displayed some caspase-3 activity that, however, was clearly less than in MCF-7/casp3-10b cells and hence, not sufficient to induce apoptosis. An even clearer picture emerged when we analyzed caspase processing in these cells in the presence and absence of CAAP. As judged by the appearance of cleavage fragments (caspase-3, -8, and -9) or by the loss of the pro-form (caspase-10), all four caspases investigated were processed following knockdown of CAAP demonstrating a caspase-dependent apoptotic pathway (Fig. 6D). Processing intensities of caspases -3, -8 and -10 appeared to be interdependent (Fig. 6D). For instance, processing of caspase-8 was dramatically accelerated in the presence of either caspase-3 or caspase-10 compared to the parent cells and was further enhanced in MCF-7/casp3-10b cells that express both caspases (Fig. 6D). This interdependency was even more pronounced for caspase-3 and -10 that were both only marginally processed when expressed individually. In MCF-7/casp3-10b cells, however, processing of both caspases increased dramatically (Fig. 6D), suggesting that caspase-3 and -10 require each other for an efficient activation process. Relative to parental cells, processing of caspase-9 was observed in all of the MCF-7 cell lines, but was the strongest in the MCF-7/casp3-10b cells. This suggests that both caspase-3 and -10 are also required for the processing of caspase-9 (Fig. 6C and 6D). It is noted that in Fig. 6D, the amount of procaspase-9 is significantly reduced in the si67 lane compared to Ncontrol lane. Together, these data are not only consistent with the caspase activation profiles obtained following knockdown of CAAP (Fig. 6C), but also with the degree of apoptosis induction that was the highest in MCF-7/casp3-10b cells (Fig. 6B).

**Figure 7:**
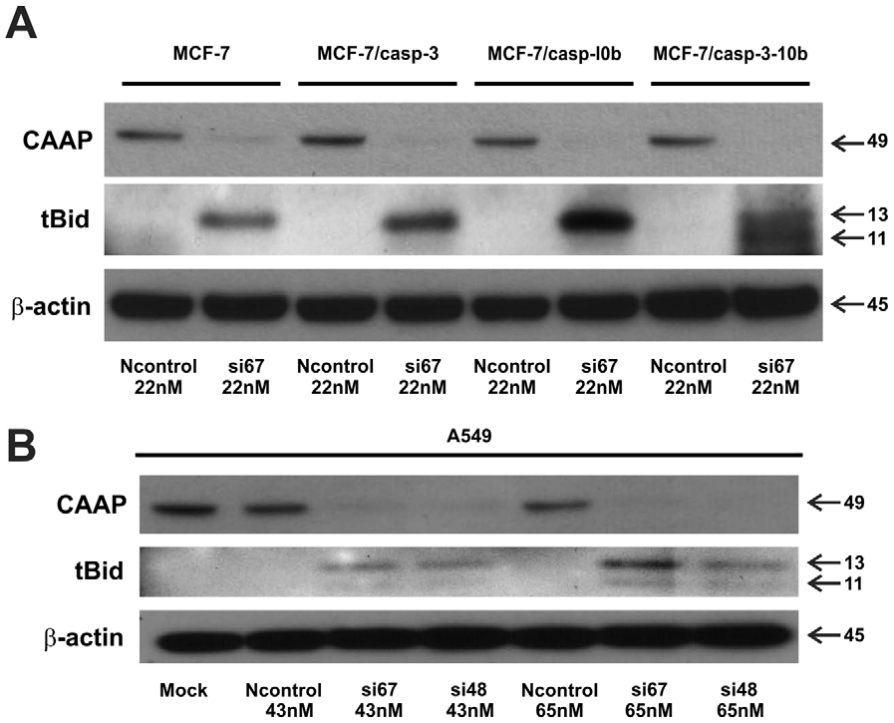
Western blot analysis of Cleavage of Bid after knockdown of CAAP in the MCF-7 cell lines and A-549 cells. (**A**) The upper panels show Bid cleavage was observed in each of the MCF-7 cell lines after knockdown of CAAP by transfection with si67 at 22 nM, but not in the Ncontrol lanes. In addition to the 13 kDa fragment, observed in each of the MCF-7 lines after knockdown of CAAP, an 11 kDa tBid fragment was detected in the MCF-7/casp3-10b cells. This is consistent with the observation that only in the MCF-7/casp3-10b cell line was significant apoptosis induced by knockdown of CAAP. (**B**) Although cleavage of Bid was observed in the treatment with both si67 and si48 transfection at both doses, the cleavage bands in the A-549 cells were weaker than in the MCF-7 cell lines.

Finally, we analyzed cleavage of Bid into tBid that can be mediated by several caspases including caspase-3, -8 and -10 [28]. Consistent with the varying levels of processed and activated capase-8 following depletion of CAAP (see Fig. 6D), cleavage of Bid into an approximately 13 kDa fragment was detected accordingly in all four MCF-7 lines (Figure 7A). However, the levels of tBid further increased upon caspase-10 expression in MCF-7 cells demonstrating that caspase-10 significantly contributes to this event. Most interestingly, whereas a larger 13 kD fragment of Bid was detected in each of the four MCF-7 cell lines, a faster migrating 11 kD tBid fragment was only generated in Caspase-3-10b cells which is the only cell line exhibiting significant apoptosis after knockdown of CAAP expression. Moreover, both tBid fragments were also detected following CAAP knockdown in caspase-10-expressing A-549 cells indicating that these events play an important role in apoptosis induced by knockdown of CAAP expression (Fig. 7B).

### Chemotherapeutic Agents Suppress CAAP Expression

**Figure 8:**
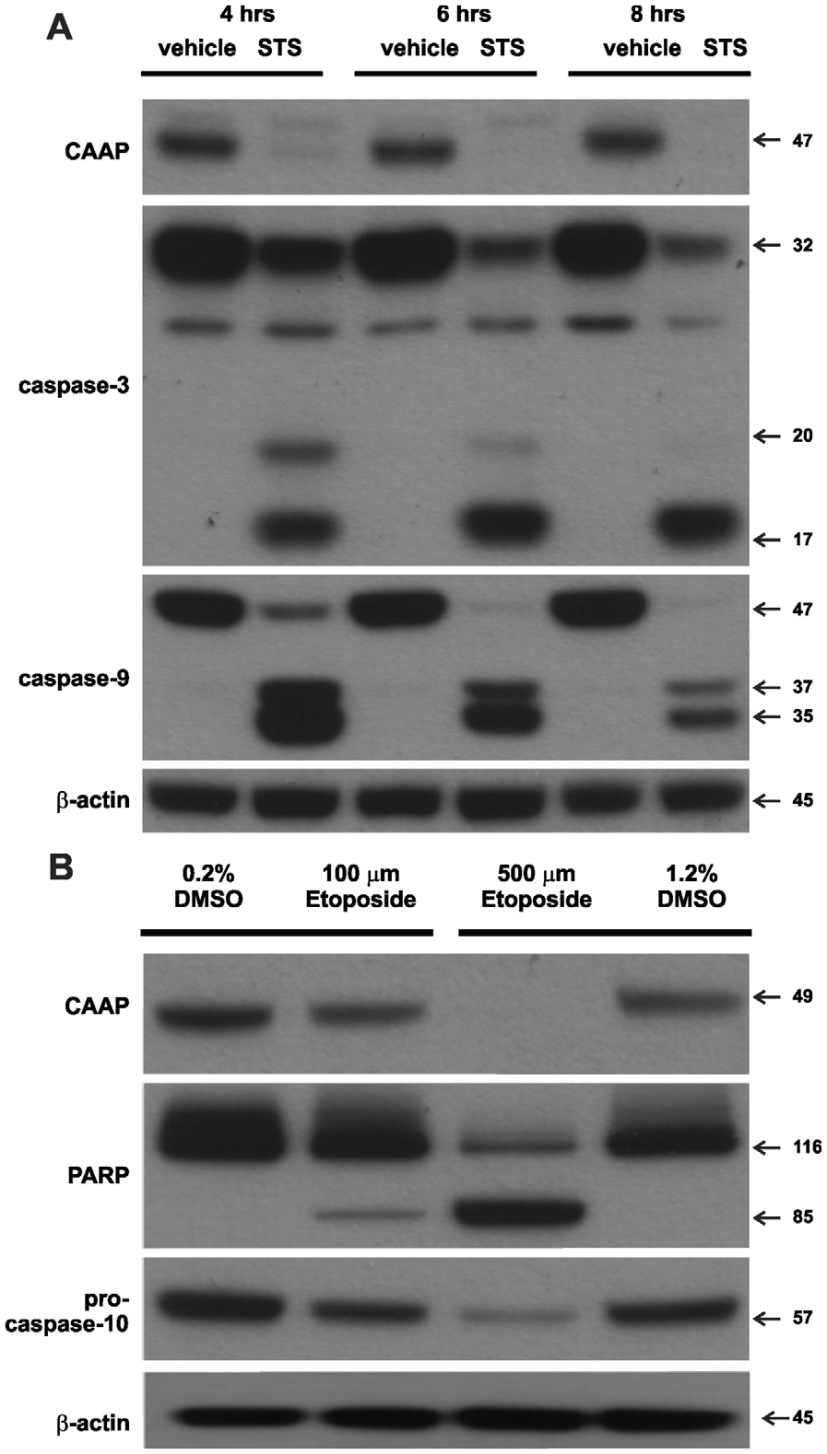
Knockdown of CAAP expression after treatment of cells with chemotherapeutic agents. (**A**) MCF-7/casp3-10b cells were treated with starurosporine (STS) for 4, 6, or 8 hrs and then examined for CAAP expression and cleavage of procaspase-3 and-9 by Western blot analysis. (**B**) MCF-7/casp3-10a cells were treated with etoposide at dose of 100 µM and 500 µM for 24 hrs and then harvested for western blot analysis of CAAP expression and cleavage of PARP and procaspase-10 expression.

Although CAAP knockdown was sufficient to initiate apoptosis, the response of CAAP to cell-extrinsic apoptotic stimuli was not known. Staurosporine (STS) is a well known chemical agent that induces apoptosis in numerous cancer cell lines [29]. We treated the MCF-7/casp3-10b cells with a low dose (1.0 µM) of STS which resulted in the knockdown of CAAP expression and induction of apoptosis based on the strong cleavage of caspase-3 and -10b for each of the three time points of treatment (Fig. 8A). Although STS is not a well known chemotherapeutic agent, there are numerous ongoing clinical trials using STS derivatives in combination with other drugs [30]–[35]. Etoposide, a widely used chemotherapeutic agent [14], was tested for effects on CAAP protein expression. As shown in Fig. 8B, the high dose of etoposide (500 µM) completely abrogated CAAP protein expression and induced apoptosis based on PARP cleavage and the processing of procaspase-10. It should be noted that the lower dose of etoposide (100 µM) had a small effect on the cleavage of PARP and processing of procaspase-10 expression. Altogether, the data suggest that CAAP is a newly discovered element of the apoptosis control pathway that includes caspases -10 and -3. Future studies will focus on determining the mechanisms and functions of CAAP regulation on apoptosis, and the cellular response to cytotoxic chemotherapeutics.

## Discussion

CAAP is expressed to varying degrees at the mRNA and protein level in every normal (adult and fetal) and cancer tissues of human origin examined and exhibit an evolutionarily preserved structure with several well-conserved domains. In particular, the central region of CAAP is predicted by bioinformatics analyses to share structural similarity with the death effector domain implying a role in apoptosis signaling. Indeed, siRNA-mediated knockdown of CAAP expression induces apoptosis in different cancer cell lines (A-549, MCF-7/casp3-10b) that proceeds independently of the caspase-8-dependent death receptor pathway, suggesting an involvement of mitochondria. This is consistent with the cleavage of Bid into tBid that was most pronounced in MCF-7/casp3/10b cells that among the various MCF-7 lines proved to be the most sensitive line toward apoptosis induction by knockdown of CAAP. In addition, together with A-549 cells, MCF-7/casp3/10b cells were the only cells in which the knockdown of CAAP resulted not only in apoptosis, but also in the generation of an 11 kDa tBid fragment. Although caspase-10 was shown, at least *in vitro*, to be responsible for this cleavage [27], its physiological impact on apoptosis signaling is unknown. Remarkably, although apoptosis induced by the knockdown of CAAP results in the processing and activation of caspassses-3, -8, -9 and -10, it requires the simultaneous activation of caspase-3 and caspase-10 to induce apoptosis. MCF-7 cells lacking caspase-10, but expressing endogenous caspase -8 and -9 do not succumb to apoptosis, even following exogenous expression of caspase-3. Only the additional presence of caspase-10 rendered MCF-7/casp3-10b cells sensitive toward apoptosis induction by the knockdown of CAAP. However, to fulfill this role, caspase-3 is required for an efficient activation of caspase-10 indicating the necessity for an interdependent caspase activation network.

Apoptosis induced by the knockdown of CAAP proceeds independently of the death receptor pathways (Fig. 5B), but requires both caspase-3 and -10. The lack of caspase-10 and/or caspase-3 significantly prevented apoptosis not only in parental MCF-7 cells, but also in the MCF-7/casp3 and MCF-7/casp-10b derivatives. The prominent role of caspase-10 is also documented by our results showing that CAAP negatively regulates either directly or indirectly the expression of procaspase-10. Thereby, it appears possible that the knockdown of CAAP results in apoptosis induction merely by a facilitated oligomerization of caspase-10 due to an increased caspase-10 pool. Also, caspases-8 and -9 are activated after knockdown of CAAP expression in all four MCF-7 clones and in A-549 cells and, this implies that CAAP may also regulate the activation of both caspase-8 and -10. Thus, we propose that CAAP is an anti-apoptotic protein which negatively regulates an apoptosis pathway that probably proceeds via activation of mitochondria as apoptosis correlated with cleavage of Bid (Fig. 7).

**Figure 9:**
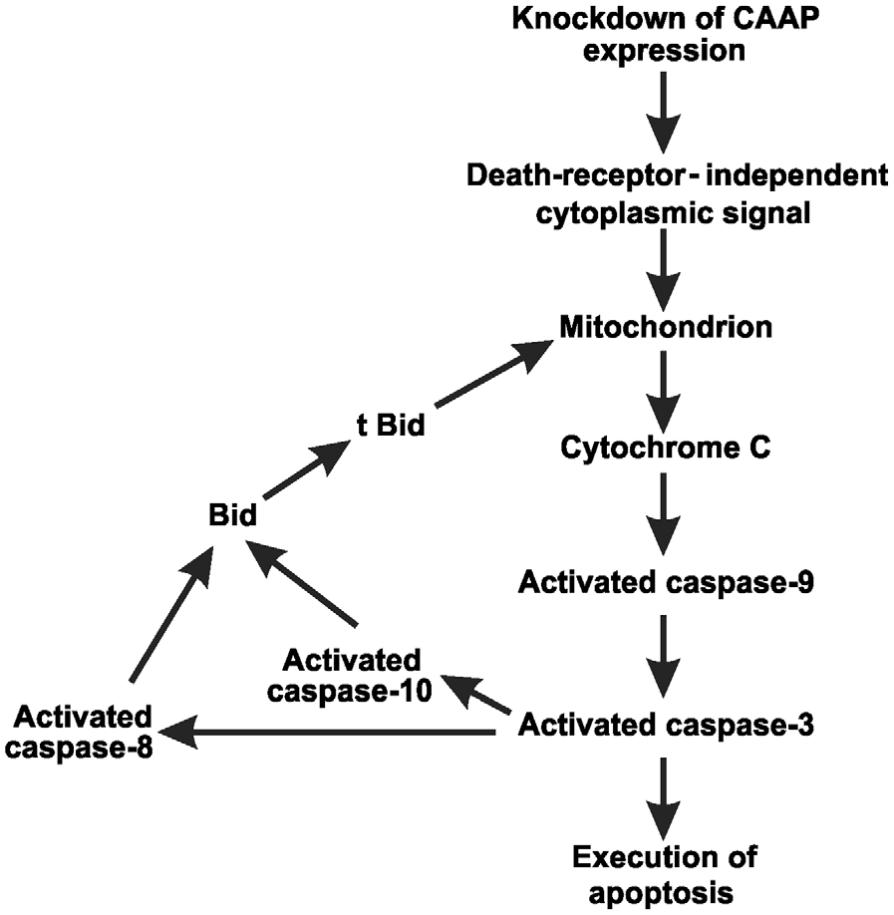
Schematic representation of Caspase-3/9 feedback loop of amplification which is induced by knockdown of CAAP expression in A-549 and MCF-7/casp3-10b cells.

The requirement for caspase-3 in the activation of caspase-10 has been previously observed [14]. Our data suggest a similar feedback amplification loop, in which caspase-10 and -8 activate caspase-9 and thus caspase-3. The lack of caspase-3 in MCF-7/casp-10b cells reduces the activity of caspase-10b by 83%, and that of caspase-8 by 75% which implies that activation of these caspases are responsive to caspase-3. Likewise, the lack of caspase-10 also reduced the activation of caspase-9 by 65% (compare caspase-9 activity in MCF-7/casp3-10b cells to its activity in MCF-7/casp3) (Fig. 6C), implying that full activation of caspase-9 requires caspase-10 and -8. Based on these data, we propose that the knockdown of CAAP induces a caspase3/9 feedback amplification loop (Fig. 9). Soo-Jung Park [4] and Ho-June Lee [36] also identified novel caspase-10 dependent apoptotic pathways. However, these pathways were not related to a feedback amplification loop (Fig. 9).

Chemotherapeutic agents also induce the mitochondrial death pathway involving an amplification loop. Filomenko et al (2006) [14] treated U937 or HeLa cells with etoposide which induced a similar caspase 3/9 amplification feedback loop. Haefen et al (2003) [16] treated BJAB cells with paclitaxel which induced a 3/8 amplification feedback loop that is also similar to the CAAP regulated amplification loop, except that this 3/8 loop does not include caspase-10, whereas CAAP controls a loop including both caspase-8 and -10 as amplifying executioners. In addition, an *in vivo* analysis of programmed cell death of dorsal root ganglia neurons in mice demonstrated that the apoptotic pathway proceeded via a 3/9 feedback amplification loop [37]. Our identification of an ubiquitously expressed and evolutionary conserved CAAP gene as a negative modulator of the 3/9 feedback amplification loop requiring caspase-10 thus represents a novel pathway that might result in the development of new therapeutic strategies.

Due to numerous p53-binding sites in its promoter region, procaspase-10 expression can be induced in a p53-dependent manner in response to DNA damage caused by chemotherapeutic agents [36]. One consequence of increased procaspase-10 is to contribute to p53-induced apoptosis following irreversible cellular damage. CAAP might also have a role in p53-induced apoptosis as it negatively modulates procaspase-10 expression and function. Interestingly, the promoter region of CAAP contains a sequence that is also present in the promoter of the retinoblastoma gene, where it constitutes a cis-acting element susceptible to negative regulation by the tumor suppressor p53 [23]. Thus, it is conceivable that p53-induced apoptosis requires prior repression of CAAP transcription.

In summary, our results demonstrate that CAAP is a ubiquitously expressed and evolutionarily conserved protein exhibiting a potent anti-apoptotic function. It appears that CAAP interferes with the activation of caspase-10, that in turn regulates the generation of an 11 kDa tBid fragment and a caspase-3/9 feedback amplification loop required for an efficient activation of the mitochondrial death pathway. Although our data suggest that CAAP restrains a caspase-3/9 feedback amplification loop that is caspase-10 dependent and utilized by some chemotherapeutic agents, the direct mechanism by which CAAP controls the expression of caspase-10 is unknown. In addition, we demonstrated that CAAP protein levels are drastically reduced in response to STS or etoposide, concurrent with the activation of apoptosis. These results suggest that CAAP may be a target for chemotherapy site since it does not require siRNA to knockdown the expression of this anti-apoptotic protein. Further studies will be required to determine the molecular mechanisms and components contributing to the anti-apoptotic functions of CAAP.

## Supporting Information

Figure S1 **Expression of CAAP and the main splice variant as assayed by RT-PCR.** Twenty PCR cycles were performed for each tissue. A human control cDNA (**Clontech**) was used in each of the 4 panels and is shown in the last Colum in each panel. Note that the expression of the control is the same in each panel. Normal tissue panel 1: heart, brain, placenta, lung, liver, kidney, pancreas. Normal tissue panel 2: spleen, thymus, prostate, testis, ovary, small intestine, colon, leukocytes. Tumor tissue panel: breast carcinoma GI-101, lung carcinoma LX-1, colon adenocarcinoma CX-1, lung carcinoma GI-117, prostatic adenocarcinoma PC3, colon adenocarcinoma GI-112, ovarian carcinoma GI-102, pancreatic adenocarcinoma GI-103. Fetal tissue panel: brain, lung, liver, kidney, heart, spleen, thymus, skeletal muscle. The last two lanes in each panel are human control cDNA (Clontech), and H_2_O control.
(TIF)

Figure S2 **Histological apoptotic phenotype of floating cells.** Light microscopy of 1 micron thick toluidine blue stained sections show that floating cells are preponderantly apoptotic after treatment with si67 and si48 at dosages of 43 nM or 65 nM. Apoptotic phenotype was found in all groups in which DNA was peripheral in the nucleus and cytoplasm was blebbing. A non-apoptotic cell is shown for comparison. Bar = 25 microns in all images.
(TIF)
